# Sterilizing Activity of Fully Oral Intermittent Regimens against *Mycobacterium Ulcerans* Infection in Mice

**DOI:** 10.1371/journal.pntd.0005066

**Published:** 2016-10-18

**Authors:** Aurélie Chauffour, Jérôme Robert, Nicolas Veziris, Alexandra Aubry, Vincent Jarlier

**Affiliations:** 1 Sorbonne Universités, UPMC Univ Paris 06, CR7, INSERM, U1135, Centre d’Immunologie et des Maladies Infectieuses, CIMI, Team E13 (Bactériologie), F-75013, Paris, France; 2 APHP, Centre National de Référence des Mycobactéries et de la Résistance des Mycobactéries aux Antituberculeux (CNR-MyRMA), Bactériologie-Hygiène, Hôpitaux Universitaires Pitié Salpêtrière-Charles Foix, F-75013, Paris, France; University of Tennessee, UNITED STATES

## Abstract

**Background:**

The treatment of Buruli ulcer (BU) that is caused by *Mycobacterium ulcerans*, is currently based on a daily administration of rifampin and streptomycin (RIF-STR). A fully oral intermittent regimen would greatly simplify its treatment on the field.

**Methodology/Principal findings:**

The objective of this study was to assess the bactericidal and sterilizing activities of intermittent oral regimens in a murine model of established *M*. *ulcerans* infection. Regimens combining rifapentine (RFP 20 mg/kg) with either moxifloxacin (MXF 200 mg/kg), clarithromycin (CLR 100 mg/kg) or bedaquiline (BDQ 25 mg/kg) were administrated twice (2/7) or three (only for RFP-CLR 3/7) times weekly during 8 weeks. The bactericidal but also the sterilizing activities of these four intermittent oral regimens were at least as good as those obtained with control weekdays regimens, i.e. RFP-CLR 5/7 or RIF-STR 5/7. A single mouse from the RFP-MFX 2/7 group had culture-positive relapse at the end of the 28 weeks following treatment completion among the 157 mice treated with one of the four intermittent regimens (40 RFP-CLR 2/7, 39 RFP-CLR 3/7, 39 RFP-MXF 2/7, 39 RFP-BDQ 2/7).

**Conclusions/Significance:**

These results open the door for a fully intermittent oral drug regimen for BU treatment avoiding intramuscular injections and facilitating supervision by health care workers.

## Author Summary

Buruli ulcer, caused by *Mycobacterium ulcerans*, is the third most frequent disease due to a bacteria of the mycobacteria family, after tuberculosis and leprosy. The disease consists of a cutaneous infection leading to skin ulcerations that can cause severe and debilitating scars. It occurs mostly in Africa where access to healthcare is still difficult. Until recently, surgery was the only way to treat Buruli ulcer. Recently, a medical treatment based on two antibiotics (rifampin and streptomycin) was found to be active in an experimental infection model and was recommended by WHO in 2004 for humans. A drawback of this new medical approach is a long and daily administration (2 months) of streptomycin, an aminoglycoside, which requires daily injections and can have secondary effects. In our research, we sought to find a fully oral and intermittent treatment to facilitate the management of the patients on the field. The antibiotics combinations we studied were efficient in our model and would need now to be tested on the field.

## Introduction

Buruli Ulcer (BU), is an infectious disease caused by *Mycobacterium ulcerans* that is mostly prevalent in Africa, but also found in Australia, Southeast Asia and South America [1]. Recently, cases have also been reported in Japan [2]. Until 2004, surgery to remove infected tissue followed by skin grafting was the only effective treatment [3] but recurrence rates ranged between 16% and 28% [4].

In 2004, the World Health Organization recommended to treat BU with a combination of rifampin (RIF) and streptomycin (STR) administered daily during 8 weeks [5]. This standard drug regimen appeared to be effective [6], well tolerated, and of low cost. However, this regimen is not fully satisfactory because it requires daily injection of STR, which is operationally demanding in most countries where BU is endemic, especially in rural areas, and exposes to aminoglycosides toxicity but also to the risk of transmission of blood-borne viral infection.

An effective, easy to organize, orally administered regimen would greatly simplify the BU treatment under field conditions. Oral regimen based on the daily administration of RIF in coordination with clarithromycin (CLR) or fluoroquinolone has been shown to be sterilizing in mice [7]. In humans, the RIF-CLR combination has been successfully used in a continuation oral phase after an initial 2 or 4-week RIF-STR phase in Ghana [8,9], as well as a fully oral treatment of a small cohort of patients with limited BU lesions in pilot studies carried out in Benin [10] and Australia [11]. Another oral regimen combining RIF and either ciprofloxacin or moxifloxacin (MXF), has been successfully used in Australia [12].

A fully oral treatment administered intermittently, e.g. twice or three times weekly, would further simplify the management of BU treatment, particularly in areas with limited access to health care facilities. In a previous study in mice, we have shown that twice-weekly administration of rifapentine (RFP), a long half-life ansamycin, in combination with streptomycin or moxifloxacin, was as bactericidal as daily administration of the corresponding regimens containing RIF in place of RFP [7]. However, the sterilizing activity of intermittent oral regimens has not been evaluated so far.

The present study aimed at evaluating, in a murine model of established infection by *M*. *ulcerans*, the sterilizing activity of fully oral intermittent (twice or three times weekly) regimens based on RFP combined with either CLR, MXF or bedaquiline (BDQ), a new antimycobacterial drug, which has been shown to be active against *M*. *ulcerans* [13,14].

## Material and methods

### Infection of mice with *M*. *ulcerans*

Four hundred and eighty 4 weeks-old female balb/c/j mice (Janvier Labs, Le Genest Saint-Isle, France), weighing around 20g, were inoculated in the left hind footpad according to the Shepard method [15] with 0.03 ml of a bacterial suspension containing 4.3 log_10_ Colony Forming Unit (CFU) of *M*. *ulcerans* strain Cu001. The strain Cu001 was isolated from a Buruli ulcer patient in Adzopé, Ivory Coast, in June 1996 [16], and was maintained in our laboratory by regular passage into mice footpads since then. The strain was kindly provided by the local laboratory without any identification data regarding the patient. This strain is susceptible to all drugs used in BU treatment.

According to the European directive 2010/63/UE, mice were held by 5 in II L cages with shaving of cellulose proposed as enrichment. Water and food were given ad libitum. A temperature of 22 +/-3°C, a hygrometry of 55 +/- 5% and a light/dark cycle 12/12 were maintained in the animal facility.

### Treatment of mice

Seven weeks after the inoculation, the infection was well established in mice as they had developed a swelling in their inoculated footpad. As previously described [17], infected footpads were scored by using a lesion index from 0 (no lesion on the inoculated footpad) to 5 (death of the mouse likely related to *M*. *ulcerans* infection). Treatment of mice began when infected footpad reached a lesion index between 2 (i.e., inflammatory swelling limited to the inoculated footpad) and 3 (i.e., inflammatory swelling involving the entire inoculated footpad).

The 480 inoculated mice were randomly allocated into eight groups (randomization.com): one untreated control group of 60 mice and seven treated groups of 60 mice each. The day of treatment initiation, 20 mice of the control group were sacrificed for CFU enumeration in the footpad in order to establish the pretreatment value (D0).

Four fully oral intermittent treatment regimens (twice or three times a week), RFP-MXF 2/7, RFP-BDQ 2/7, RFP-CLR 2/7 and RFP-CLR 3/7, as well as the intermittent RFP-STR 2/7, were compared to reference regimens, the oral regimen RFP-CLR 5/7 [7] and RIF-STR 5/7 [18].

Treatment began immediately after randomization. RIF was purchased from Sandoz laboratory, France; MXF from Bayer Santé, France; CLR from Abbott France, France; STR from Sigma, France; RFP from Sequoia Research, United Kingdom; and BDQ was kindly provided by Janssen Pharmaceutica, Belgium. Dosages were as follows: RIF 10 mg/kg; MXF 200 mg/kg; CLR 100 mg/kg; RFP 20 mg/kg when administered twice a week and 10 mg/kg when administered 5 times per week; STR 150 mg/kg; and BDQ 25 mg/kg. RIF, MXF, RFP and CLR were re-suspended in 0.05% agar-distilled water; STR was diluted in normal saline; and BDQ was directly provided by Janssen Pharmaceutica in a 20% hydropropyl-β-cyclodextrin formulation. All drugs were orally administered by gavage in a volume of 0.2 ml except for STR, which was injected subcutaneously in the same volume.

Ten mice per group were sacrificed after 4 weeks of treatment and ten others after 8 weeks of treatment. The other 40 mice from each group that had been treated during 8 weeks were held without treatment and observed during an additional 28-week period to monitor relapses of *M*. *ulcerans* infection.

Mice were sacrificed by cervical dislocation as recommended by the French decree n°2013–118 and the European directive 2010/63.

### Assessment of severity of *M*. *ulcerans* infection and of treatment effectiveness

The severity of the infection and the effectiveness of treatment were assessed in the different groups (i) clinically by measuring the lesion index, and (ii) bacteriologically by cultivating *M*. *ulcerans* on an appropriate medium to get the mean number of CFU per inoculated footpad after 4 and 8 weeks of treatment. The sterilizing activity of the treatment was assessed by (i) measuring the lesion index during the 28-week observational period and (ii) culturing the footpad at the end of this period.

For CFU enumeration, the tissues from the footpad were removed aseptically and homogenized in a Hank’s balanced salt solution in a final volume of 2 ml. Suspensions were then plated onto Lowenstein-Jensen medium. For untreated control groups, suspensions were serially diluted in 10-fold steps (from pure to 10^−4^) and plated in duplicate onto the medium with 0.1 ml of the diluted suspension. For the treated groups, the entire volume of the footpad suspension was plated onto 10 tubes of the medium each with 0.2 ml of the suspension. All tubes were incubated at 30°C up to 90 days.

Mice were examined weekly to assess the evolution of the lesion index in order to monitor the relapse during the post-treatment follow-up period. A rebound of the lesion index to ≥3 of the inoculated footpad suggested clinical relapse and led to immediate sacrifice for footpad culture. For that purpose, the entire volume of the footpad suspension was cultivated onto Lowenstein Jensen medium as described above. A culture positive for *M*. *ulcerans* within 90 days of incubation was taken as a confirmation of relapse. At the end of the 28 weeks of observation following the end of the treatment, all the remaining mice were systematically sacrificed and their footpads were cultivated as described above. A positive culture at the end of the incubation time was considered to be a culture-positive relapse.

### MIC determination

In order to assess a possible acquisition of resistance to the antibiotics used during treatment, the bacilli cultivated from relapsing mice (during the observation period) were tested for *in vitro* susceptibility and molecular detection of mutation implicated in antibiotic resistance.

The bacilli cultivated from relapsing mice as well as the susceptible strain Cu001, were re-suspended in distilled water and the turbidity was adjusted to Mac Farland 3 (1 mg/ml). RIF, RFP, MXF and CLR were tested on a 7H11 + 10% OADC (Oleic-Acid-Dextrose-Catalase) medium (pH 7.4). CLR has also been tested on MH media (pH 6.6). RIF and RFP were dissolved in dimethylformamide, and MXF and CLR in distilled water to obtain a final concentration of 4 μg/ml in medium. They were then twofold diluted in their own solvent and incorporated to the culture media to obtain a final range from 4 to 0.12 μg/ml. 0.1 ml of 2 bacilli suspensions (pure and 10^−2^) were plated onto drug-containing media and drug-free media used as a growth control. All media were incubated at 30°C and examined after 60 and 90 days [13,19].

### Molecular detection of selection of antibiotic resistance

A part of the bacterial suspension from relapsing mice was inactivated at 95°C during 30 min, to allow bacterial DNA extraction by heat shock (5 cycles of 2 min at 95°C and 2 min at 4°C) following by 15 min at 95°C and 15 min in an ultrasonic bath.

IllustraTM PuReTaq Ready-To-Go PCR beads (GE Healthcare) was used to perform PCRs. For the *rpoB* gene, we used primers as previously reported [17] RPOBMuS 5’-GCGCACGGTGGGTGAGCTG-3’ RPOBMuAS 5’-CGAGACGCCCTACCGCAAGG-3’. Other primers were designed according to information on the complete genome of *M*. *ulcerans* (GenBank accession number CP000325): for the *gyrA* gene, MugyrAS 5’-CGCCGTGTGCTCTATGCCATG-3’ and MugyrAAS TCGCCGGGTAATGACCCGCCA-3’, and for the *ARN23S* gene 23.1, 5’-AATGGCGTAACGACTTCTCAACTGT-3’ and 23.2 5’-GCACTAGAGGTTCGTCCGTCCC-3’.

PCR-amplified fragment were purified by using Qiagen DNA purification kit (Qiagen) and sequenced by the dideoxychain termination method with the ABI PRISM BigDye Terminator V3.1 Cycle Sequencing Kit (Life Technologies). The oligonulceotide primers used for DNA sequencing were the same as those used for PCR. The sequences were analyzed with the software Seqscape 2.0 (Life Technologies).

### Statistical analysis

The Student’s t test and the Fisher exact test were used for groups’ comparison. A p-value <0.05 was considered as statistically significant. A regimen was considered to be bactericidal if its mean value of CFU per footpad was significantly lower than in the untreated group.

### Ethic statement

Our animal facility received in April, 24^th^ 2012 the authorization to carry out animal experiments (license number B-75-13-01). The persons who carried out the animal experiments had a personal authorization delivered by the Direction Départementale de la Protection des Populations de Paris. We followed the experimental guidelines of the Faculté de Médecine Pierre et Marie-Curie to carry out the experimental project.

## Results

### Average lesion index and CFU counts during the treatment phase

**Fig 1:**
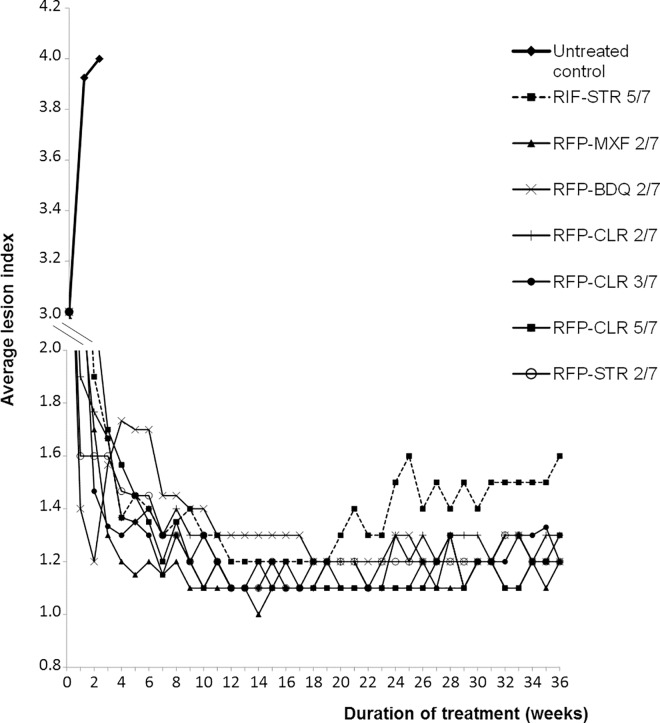
Evolution of the average lesion indexes in the control and treated groups.

The evolution of the footpads swelling is shown in Fig 1. Inoculated footpads of the untreated control mice had swollen from a mean lesion index 3 at D0 (beginning of treatment) to index 4 at week 2, where mice were sacrificed before predictable death due to *M*. *ulcerans* infection and to avoid culture contamination. In all treated mice, footpad lesions drastically decreased from a mean index 3 at D0 to mean indexes ranging from 1.2 (RFP-MXF 2/7 group) to 1.6 (RFP-CLR 5/7 group) after 4 weeks of treatment. During the next 4 weeks of treatment (i.e. till completion of 8 weeks treatment), the mean lesion indexes remained globally stable ranging from 1.2 (RFP-MXF 2/7 group) to 1.5 (RFP-BDQ 2/7 group).

The results of the footpad CFU counts are shown in Table 1. All untreated mice had culture-positive footpads at D0 (beginning of treatment) and the mean number of CFU per footpad had increased from 6.39±0.30 to 6.63±0.29 log_10_ at week 4.

**Table 1:** Results of footpad cultures during the treatment of mice infected with *M*. *ulcerans* and relapse rate after treatment completion.

Group	Treatment^a^	Results during treatments						Follow-up period (28 weeks after treatment completion)		
		Day 0		4 weeks		8 weeks				
		Culture positivity rate	Mean (±SD) CFU per group	Culture positivity rate	Mean (±SD) CFU per group	Culture positivity rate	Mean (±SD) CFU per group	Culture positivity rate	Mean (±SD) CFU per group	Interval (weeks) between end of treatment and relapse
**Control groups**	**Untreated**	20/20	6.39±0.30	40/40	6.63±0.29^b^					
	**RIF-STR 5/7**			10/10	0.73±0.65	3/10	0.14±0.34	4/40	0.33±1.00	12, 13, 16, 17
	**RFP**_**10**_**-CLR 5/7**			1/10	^c^	0/10		1/40	0.08±0.52^c^	28
**Test groups**	**RFP**_**20**_**-STR 2/7**			2/10	0.16±0.42	0/10		0/40		
	**RFP**_**20**_**-CLR 2/7**			2/10	0.07±0.22	0/10		0/40		
	**RFP**_**20**_**-CLR 3/7**			1/10	0.08±0.24	0/10		0/39		
	**RFP**_**20**_**-MXF 2/7**			3/10	0.19±0.34	0/10		1/39	0.07±0.44	28
	**RFP**_**20**_**-BDQ 2/7**			3/10	0.19±0.42	0/10		0/39		

^a^ Treatments began 7 weeks after inoculation with 4.3 log_10_ CFU per footpad when the infected swelling footpads reached a lesion index between 2 and 3. Drugs were administered 2, 3 or 5 times per week depending on the group. The dosages were as follows: RIF 10 mg/kg body weight; MXF 200 mg/kg body weight; CLR 100 mg/kg body weight; RFP 20 mg/kg body weight when administered 2/7 and 10 mg/kg when administered 5/7; STR 150 mg/kg body weight; and BDQ 25 mg/kg body weight.

^b^ due to advanced lesion index and to avoid culture contamination, all untreated control mice were sacrificed at 4 weeks

^c^: only one CFU recovered from this mouse

After 4 weeks of treatment with the RIF-STR 5/7 reference regimen, all mice remained culture positive, although the mean CFU counts dramatically decreased to reach <1 log_10_. In the RFP-CLR 5/7 reference group, as well as in all test groups, the mean CFU was constantly <1 log_10_.

After 8 weeks of treatment, mice were culture negative in all treated group, except in the RIF-STR 5/7 reference group where three mice still had positive footpads with only 1, 2 and 12 colonies, respectively, recovered from these entire footpads. The proportion of culture-negative mice at 8 weeks, as well as the mean CFU count per footpad were not statistically significant among all groups including the RIF-STR 5/7 group (p>0.05).

### *M*. *ulcerans* infection relapses during the follow-up phase

During the 28 weeks of follow-up after treatment completion, 4 mice of the RIF-STR 5/7 group exhibited a rebound of their footpad lesion and were also culture-positive, confirming relapses. These relapses occurred between 12 and 17 weeks after the end of treatment. At the end of the 28-week follow-up, all mice were sacrificed for CFU determination. Concerning the RFP_10_-CLR 5/7, 1/40 mice were culture-positive whereas among the 5 oral intermittent test groups, only one (1/39 in the RFP-MXF 2/7 group) mouse was culture-positive among the 157 observed mice.

Compared to the STR-RIF 5/7 group, the mean CFU count at the end of the follow-up in the RFP-CLR 5/7 group and in the RFP-MXF 2/7 group was not statistically significant (p = 0.17 and 0.16, respectively). On the opposite, all other intermittent regimen had significantly lower mean CFU count than the STR-RIF 5/7 group (p = 0.04 for all comparisons).

### Bacteriological analysis of *M*. *ulcerans* bacilli recovered from relapsing mice

Initial antibiotic MICs against *M*. *ulcerans* Cu001 reference strain were in the susceptible range: 0.5μg/ml for RIF, 0.5–1 μg/ml for RFP, 0.25–0.5 μg/ml for MXF, 0.5 μg/ml for STR, and 0.5 μg/ml for CLR (same value on both 7H11 and MH media). MICs remained unchanged for all drugs against bacilli grown from relapsing mice in orally treated groups, and results were as follow: RIF 0.5–1 μg/ml, RFP 0.5μg/ml and MXF 0.5 μg/ml against bacilli from the sole positive mouse in the RFP_20_-MXF 2/7 group; RIF 0.5–1 μg/ml, RFP 0.5–1 μg/ml and CLR 0.5 μg/ml (on 7H11) and 0.5–1 μg/ml (on MH) against bacilli from the sole positive mouse in the RFP_10_-CLR 5/7 group. The results were similar for bacilli isolated from relapsing mice treated with RIF-STR 5/7.

Finally, no mutations conferring resistance were detected by molecular biology in bacilli grown from relapsing mice.

## Discussion

The bactericidal activity of an intermittent 8-week treatment regimen with 2/7 administration of RFP 20 mg/kg in combination with either STR or MXF has been previously reported, raising the hope that Buruli ulcer might be treated with twice-weekly therapy [7]. However, the sterilizing activity, i.e. the lack of relapse after treatment completion, of the RFP-MXF 2/7 oral intermittent regimen was unknown. The present study showed that three fully oral intermittent regimens, based on rifapentine combined with moxifloxacin, clarithromycin, on the one hand, or bedaquiline, on the other hand, were highly bactericidal after 8 weeks of treatment but also had sterilizing activity against *M*. *ulcerans* infection in mice.

The RIF-STR 5/7 regimen, which is the main reference drug regimen used for treating BU in endemic countries, was bactericidal. However, it lacks of sterilizing effect when compared to the other regimens in the present experiment, although the differences did not reach statistical significance (p>0.05). This lack of sterilizing activity has been already reported in a previous study where the reduction in the RIF-STR dosing frequency from 7/7 to 5/7 led to a relapse rate of 12% in the mouse model [18].

In the present study, the intermittent 2/7 or 3/7 fully oral regimens were as active as the control oral regimen (RFP-CLR 5/7). We confirm by the present study that the latter regimen is sterilizing in the mouse model (a single relapse among 40 mice), as previously found in the same model of established infection [7] as well as in a model of incubating infection [20]. Therefore, the RFP-CLR 5/7 regimen is worth comparing to the daily RIF-CLR oral regimen, which has been successfully tested in the field [10] and is currently evaluated in a clinical trial (NCT01659437, clinicaltrials.gov) under the supervision of WHO. We did not test RIF-CLR intermittent regimens in order to compare RIF and RFP activities when combined to CLR. However, we have already reported the lack of sterilizing effect of RIF-STR intermittent regimens [18]. This previous result together with the present experiment suggest that the difference is due to the RFP effect, and likely to its long half-life.

We report here that three fully oral intermittent regimens were sterilizing in the BU mouse model. The combinations using CLR instead of MXF have the potential interest to be used in children, who represent a high proportion of BU patients. Of note, in a case report from Australia [21], the use of a RIF-CLR intermittent regimen has been successful for treating a child. In addition, CLR is currently under investigation in a clinical trial of BU in Ghana and Benin [10]. The use of BDQ in the treatment of BU has already been investigated in the mouse model [13], and has shown to be sterilizing when used daily with RIF. Here, we demonstrated its sterilizing activity when administered 2/7 combined with a long half-life ansamycin, RFP.

Intermittent administration of combined regimen raises the question of pharmacokinetic mismatch between the drugs combined in the regimen. A large mismatch will result in effective monotherapy and potentially favor the selection of resistant mutants. The half-lives of MXF and BDQ are very close to that of RFP, thus 2/7 administration of RFP-MXF or RFP-BDQ should be effective in preventing the selection of ansamycin-resistant mutants. The half-life of clarithromycin in humans is shorter (4–5 hours) than that of rifapentine (around 14–16 hours). Therefore, this discrepancy between the two half-lives suggests using the RMP-CLR combination three times weekly rather than twice weekly, a rhythm that can still coincide with visits to health care centers.

The 20 mg/kg RFP dosage used in the present experiment was higher than the classical 10 mg/kg dosage used for daily administration. The higher dosage was justified for a twice-weekly administration since it aims at counterbalancing the lower number of administration. High RFP dosages have been used in the treatment of tuberculosis in humans and were well tolerated when administrated weekly [22–24]. Moreover, BU occurs very often in children and higher weight-normalized RFP dosages are required to obtain kinetic properties equivalent to those seen in adults [25].

The present study was conducted by using a single *M*. *ulcerans* strain. MICs of the different antibiotic against this strain are in the middle range of what was previously reported [13] and therefore the present results are likely to be valid for a majority of strains. However, treatment outcome may vary if antibiotic MICs are on the higher range. In our study, data on pharmacokinetic parameters of antibiotics used in treatment regimens were not performed, and would have helped in interpreting bacteriological results. Nevertheless, the choice of drug concentrations in the experimental model was based on data of previous studies performed in mice and derived from human pharmacokinetic results [22–24].

The results of the present work are encouraging and suggest the possibility to combine two advantages over the actual reference RIF-STR daily regimen (i) oral administration that would avoid injections and (ii) intermittent administration that would facilitate the supervision in the field. Among the intermittent oral regimens that have shown sterilizing activity, the RFP-CLR combinations are likely to be the most interesting to be tested in the field because CLR is already used in the BU treatment especially in children. In addition, the use of MXF and BDQ for the treatment of BU in countries with high tuberculosis prevalence is questionable since both drugs are essential for the treatment of multidrug resistant tuberculosis.

## References

[1] World Health Organization. Buruli ulcer: progress report, 2004–2008. Wkly Epidemiol Rec. 2008;83: 145–156. 18437758

[2] Nakanaga K, Hoshino Y, Yotsu RR, Makino M, Ishii N. Nineteen Cases of Buruli Ulcer Diagnosed in Japan from 1980 to 2010. J Clin Microbiol. 2011;49: 3829–3836. 10.1128/JCM.00783-11 21880966 PMC3209082

[3] van der Werf TS, van der Graaf WT, Tappero JW, Asiedu K. Mycobacterium ulcerans infection. Lancet. 1999;354: 1013–1018. 10.1016/S0140-6736(99)01156-3 10501380

[4] Kibadi K, Mputu-Yamba JB, Mokassa B, Panda M, Muyembe-Tamfum JJ. [Relapse after surgical treatment of mycobacterium ulcerans infection (buruli ulcer): study of risk factors in 84 patients in the Democratic Republic of the Congo]. Médecine Trop Rev Corps Santé Colon. 2009;69: 471–474. 20025176

[5] WHO | Provisional guidance on the role of specific antibiotics in the management of *Mycobacterium ulcerans* disease (Buruli ulcer). In: WHO [Internet]. [cited 17 Apr 2014]. Available: http://www.who.int/buruli/information/antibiotics/en/

[6] Sarfo FS, Phillips R, Asiedu K, Ampadu E, Bobi N, Adentwe E, et al Clinical Efficacy of Combination of Rifampin and Streptomycin for Treatment of Mycobacterium ulcerans Disease. Antimicrob Agents Chemother. 2010;54: 3678–3685. 10.1128/AAC.00299-10 20566765 PMC2935024

[7] Ji B, Chauffour A, Robert J, Jarlier V. Bactericidal and Sterilizing Activities of Several Orally Administered Combined Regimens against Mycobacterium ulcerans in Mice. Antimicrob Agents Chemother. 2008;52: 1912–1916. 10.1128/AAC.00193-08 18391038 PMC2415803

[8] Nienhuis WA, Stienstra Y, Thompson WA, Awuah PC, Abass KM, Tuah W, et al Antimicrobial treatment for early, limited Mycobacterium ulcerans infection: a randomised controlled trial. Lancet Lond Engl. 2010;375: 664–672. 10.1016/S0140-6736(09)61962-0 20137805

[9] Phillips RO, Sarfo FS, Abass MK, Abotsi J, Wilson T, Forson M, et al Clinical and Bacteriological Efficacy of Rifampin-Streptomycin Combination for Two Weeks followed by Rifampin and Clarithromycin for Six Weeks for Treatment of Mycobacterium ulcerans Disease. Antimicrob Agents Chemother. 2014;58: 1161–1166. 10.1128/AAC.02165-13 24323473 PMC3910847

[10] Chauty A, Ardant M-F, Marsollier L, Pluschke G, Landier J, Adeye A, et al Oral treatment for Mycobacterium ulcerans infection: results from a pilot study in Benin. Clin Infect Dis. 2011;52: 94–96. 10.1093/cid/ciq072 21148526

[11] Friedman ND, Athan E, Hughes AJ, Khajehnoori M, McDonald A, Callan P, et al Mycobacterium ulcerans Disease: Experience with Primary Oral Medical Therapy in an Australian Cohort. Vinetz JM, editor. PLoS Negl Trop Dis. 2013;7: e2315 10.1371/journal.pntd.0002315 23875050 PMC3715400

[12] O’Brien DP, McDonald A, Callan P, Robson M, Friedman ND, Hughes A, et al Successful Outcomes with Oral Fluoroquinolones Combined with Rifampicin in the Treatment of Mycobacterium ulcerans: An Observational Cohort Study. Johnson C, editor. PLoS Negl Trop Dis. 2012;6: e1473 10.1371/journal.pntd.0001473 22272368 PMC3260310

[13] Ji B, Lefrancois S, Robert J, Chauffour A, Truffot C, Jarlier V. In Vitro and In Vivo Activities of Rifampin, Streptomycin, Amikacin, Moxifloxacin, R207910, Linezolid, and PA-824 against Mycobacterium ulcerans. Antimicrob Agents Chemother. 2006;50: 1921–1926. 10.1128/AAC.00052-06 16723546 PMC1479135

[14] Lounis N, Veziris N, Chauffour A, Truffot-Pernot C, Andries K, Jarlier V. Combinations of R207910 with drugs used to treat multidrug-resistant tuberculosis have the potential to shorten treatment duration. Antimicrob Agents Chemother. 2006;50: 3543–3547. 10.1128/AAC.00766-06 16954317 PMC1635167

[15] Shepard CC. The experimental disease that follows the injection of human leprosy bacilli into foot-pads of mice. J Exp Med. 1960;112: 445–454. 10.1084/jem.112.3.445 19867175 PMC2137235

[16] Dega H, Bentoucha A, Robert J, Jarlier V, Grosset J. Bactericidal Activity of Rifampin-Amikacin against Mycobacterium ulcerans in Mice. Antimicrob Agents Chemother. 2002;46: 3193–3196. 10.1128/aac.46.10.3193-3196.2002 12234844 PMC128793

[17] Lefrancois S, Robert J, Chauffour A, Ji B, Jarlier V. Curing Mycobacterium ulcerans Infection in Mice with a Combination of Rifampin-Streptomycin or Rifampin-Amikacin. Antimicrob Agents Chemother. 2007;51: 645–650. 10.1128/AAC.00821-06 17101676 PMC1797726

[18] Ji B, Chauffour A, Aubry A, Robert J, Ibrahim M, Jarlier V. Impacts of dosing frequency of the combination rifampin-streptomycin on its bactericidal and sterilizing activities against Mycobacterium ulcerans in mice. Antimicrob Agents Chemother. 2009;53: 2955–2959. 10.1128/AAC.00011-09 19364857 PMC2704658

[19] Portaels F, Traore H, De Ridder K, Meyers WM. In vitro susceptibility of Mycobacterium ulcerans to clarithromycin. Antimicrob Agents Chemother. 1998;42: 2070–2073. 9687409 10.1128/aac.42.8.2070 PMC105863

[20] Almeida D, Converse PJ, Ahmad Z, Dooley KE, Nuermberger EL, Grosset JH. Activities of rifampin, Rifapentine and clarithromycin alone and in combination against mycobacterium ulcerans disease in mice. PLoS Negl Trop Dis. 2011;5: e933 10.1371/journal.pntd.0000933 21245920 PMC3014976

[21] Gordon CL, Buntine JA, Hayman JA, Lavender CJ, Fyfe JAM, Hosking P, et al All-Oral Antibiotic Treatment for Buruli Ulcer: A Report of Four Patients. Franco-Paredes C, editor. PLoS Negl Trop Dis. 2010;4: e770 10.1371/journal.pntd.0000770 21152060 PMC2994921

[22] Bock NN, Sterling TR, Hamilton CD, Pachucki C, Wang Y-C, Conwell DS, et al A Prospective, Randomized, Double-Blind Study of the Tolerability of Rifapentine 600, 900, and 1,200 mg Plus Isoniazid in the Continuation Phase of Tuberculosis Treatment. Am J Respir Crit Care Med. 2002;165: 1526–1530. 10.1164/rccm.200201-047OC 12045127

[23] Schechter M, Zajdenverg R, Falco G, Barnes GL, Faulhaber JC, Coberly JS, et al Weekly Rifapentine/Isoniazid or Daily Rifampin/Pyrazinamide for Latent Tuberculosis in Household Contacts. Am J Respir Crit Care Med. 2006;173: 922–926. 10.1164/rccm.200512-1953OC 16474028 PMC2662911

[24] Conde MB, Mello FCQ, Duarte RS, Cavalcante SC, Rolla V, Dalcolmo M, et al A Phase 2 Randomized Trial of a Rifapentine plus Moxifloxacin-Based Regimen for Treatment of Pulmonary Tuberculosis. Hatherill M, editor. PLOS ONE. 2016;11: e0154778 10.1371/journal.pone.0154778 27159505 PMC4861335

[25] Blake MJ, Abdel-Rahman SM, Jacobs RF, Lowery NK, Sterling TR, Kearns GL. Pharmacokinetics of rifapentine in children. Pediatr Infect Dis J. 2006;25: 405–409. 10.1097/01.inf.0000214963.55217.9c 16645503

